# Creatinine Deiminase Adsorption onto Silicalite-Modified pH-FET for Creation of New Creatinine-Sensitive Biosensor

**DOI:** 10.1186/s11671-016-1386-9

**Published:** 2016-03-31

**Authors:** Svitlana V. Marchenko, Oleksandr O. Soldatkin, Berna Ozansoy Kasap, Burcu Akata Kurc, Alexei P. Soldatkin, Sergei V. Dzyadevych

**Affiliations:** Laboratory of Biomolecular Electronics, Institute of Molecular Biology and Genetics, National Academy of Sciences of Ukraine, 150 Zabolotnogo Str., 03680 Kyiv, Ukraine; Institute of High Technologies, Taras Shevchenko National University of Kyiv, 64 Volodymyrska St., Kyiv, 01003 Ukraine; Micro and Nanotechnology Department, Middle East Technical University, Ankara, 06531 Turkey; Central Laboratory, Middle East Technical University, Ankara, 06531 Turkey

**Keywords:** Biosensor, Creatinine, Silicalite, pH-sensitive field-effect transistor

## Abstract

In the work, silicalite particles were used for the surface modification of pH-sensitive field-effect transistors (pH-FETs) with the purpose of developing new creatinine-sensitive biosensor. Creatinine deiminase (CD) adsorbed on the surface of silicalite-coated pH-FET served as a bioselective membrane. The biosensor based on CD immobilized in glutaraldehyde vapor (GA) was taken as control. The creatinine-sensitive biosensor obtained by adsorption on silicalite was shown to have better analytical characteristics (two- to threefold increased sensitivity to creatinine, three- to fourfold lesser response and recovery times, a decrease of the detection limit of creatinine determination to 5 μM, etc.).

Additionally, the biosensor based on CD adsorbed on silicalite (Sil/CD) was characterized by high signal reproducibility (relative standard deviation (RSD) for creatinine measurement = 2.6 %) and stability during storage (over 13 months). It was experimentally confirmed that the proposed biosensor was not sensitive either to high concentrations of sodium chloride or to the macromolecular protein fractions and can be used for direct quantitative analysis of creatinine in the blood serum.

It was concluded that the method of CD adsorption on silicalite is well-suited for the creation of creatinine-sensitive biosensor with improved working characteristics.

## Background

Zeolites are minerals of aqueous aluminosilicate group of alkali and alkaline-earth metals, which have complex three-dimensional lattice and highly ordered structure. They can be both natural and artificially synthesized [1]. An important characteristic of zeolites is the Si/Al ratio [2]. It can be changed, thus varying the zeolite charge and hydrophobicity, the number and size of pores. These properties make zeolite an important material in petrochemistry, environmental science, agriculture, medicine, and many other fields. The chemical composition of zeolites can be represented by the formula M*x*/*n*|·[(AlO_2_)*x*·(SiO_2_)*y*]·*z*H_2_O, where M is the cations of valence *n* (commonly Na^+^, K^+^, Ca^2+^, Ba^2+^, Sr^2+^, and Mg^2+^), *z* is the number of adsorbed water molecules and the ratio *у*/*х* can vary in a wide range from 1—for low-silica zeolites A and X, to ∞—for crystalline silicalites. For the latter, the cation concentration is close to zero.

Also, important characteristics of zeolites are low cost of their extraction, availability in large amounts, tunable surface properties, mechanical, and chemical resistance. They are stable in wet and dry conditions and well-tolerated by microorganisms. Nowadays, zeolites are of interest for immobilization of protein [2, 3] and especially of different enzymes due to high surface area, rigid and well-defined pore structures, thermal stability, and hydrophilicity [4]. The enzyme adsorption on zeolites is a mild and non-toxic method of immobilization, which allows retention of the enzyme activity.

An idea of enzyme adsorption on zeolites has been proposed in previous work [5]. Currently, a number of biosensors based on various types of zeolites have been developed using different enzyme systems: urease [6–11], glucose oxidase [12–14], acetylcholinesterase [15], butyrylcholinesterase [16, 17], invertase/mutarotase/glucose oxidase [18], etc.

This work was aimed at the development of a creatinine-sensitive biosensor based on pH-sensitive field-effect transistors (pH-FETs) using silicalite as an adsorbent for the creatinine deiminase immobilization (Sil/CD). Silicalite, one of the most studied zeolites, is known by its high adsorption capacity, hydrophobic, and organophilic selectivity. The development of a new method of express analysis of creatinine concentration is of vital importance because creatinine is a marker of kidney glomerular filtration rate [19, 20] and considered as a general diagnostic indicator of kidney function. Additionally, determination of creatinine concentration is essential at diabetic nephropathy, acute myocardial infarction, and for monitoring hemodialysis patients [21].

Therefore, this work was focused on silicalite application for the creation of a new biosensor for creatinine determination in model and real samples.

## Methods

### Materials

In the work, microbial enzyme creatinine deiminase (EC 3.5.4.21) with activity of 36 U/mg was purchased from Sigma-Aldrich (Japan); bovine serum albumin (BSA) (fraction V), 25 % aqueous solution of glutaraldehyde, and creatinine were purchased from “Sigma–Aldrich Chemie” (Germany); DEAE-Dextran—from Fluka Biochemica (France); and lactitol—from Fluka (Switzerland). High molecular alcohol lactitol and DEAE-Dextran were used as the enzyme stabilizers; the latter also provides retention of the enzyme molecules in the membrane and prevents their washout. The working phosphate buffer (KH_2_PO_4_-NaOH), pH 7.4, was prepared from reagents from Helicon (Moscow, Russia). Other non-organic compounds used were of analytical grade.

### Potentiometric Transducers

Potentiometric transducers based on pH-sensitive field-effect transistors were produced at the JSC “Kwazar” facilities (Kiev, Ukraine). Each transducer contained a differential pair of two identical p-channel field-effect transistors placed on a single crystal with the total area of 8 mm × 8 mm. N-type (100) silicon wafer was used as a substrate. The gate of dielectric layer was formed from thermally oxidized SiO_2_ film 50 nm thick and Si_3_N_4_ film of the same thickness deposited in the low-pressure reactor. The gate area had zigzag-shaped geometry with the length to width ratio of 100, which provided sufficient gain factor of p-channel transistors. The p-type conducting busses covered with a dielectric layer were used to form the electric contact to the transistor drain and source areas. The crystal with FETs was mounted on the specifically designed printed-circuit boards (5 cm × 0.8 cm) for convenient connection. There were two pH-FETs on each chip, which allows measurements in differential mode to avoid an influence of the non-specific changes in output signal associated with the fluctuations in temperature, environmental pH, and electrical noise. The contact from the chip to the board copper layer was made by ultrasonic welding with an epoxy glue sealing. The signals from both transistors were recorded, and then, the signal from the reference transistor (covered with the “blank” membrane) was subtracted from the signal of the transistor covered with the enzymatic membrane (biorecognition element). The transistors demonstrated pH-sensitivity of approximately 40 mV/pH and transconductance of 400–500 μA/V, thus providing pH-sensitivity of the transistor channel current of 15–20 μA/pH.

More information about the transducer structure can be found in [22] and description of the portable measuring device in [23].

### Preparation of Silicalite-Modified Electrode

**Fig. 1:**
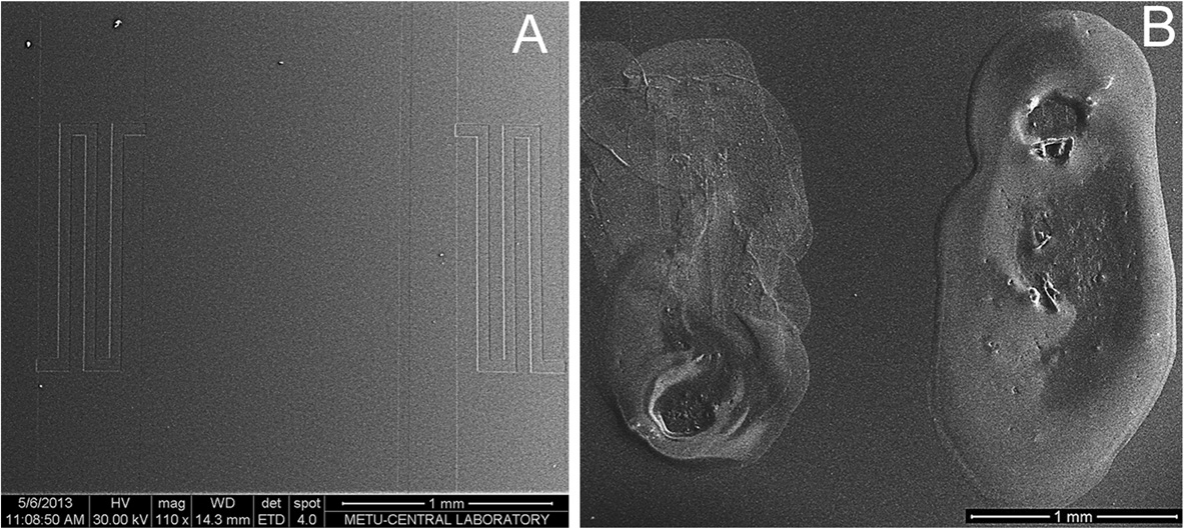
SEM images of potentiometric transducers (sensitive parts): bare surface (**a**) and surface covered with silicalite (**b**)

A silicalite layer on the transducer surface was formed by drop-coating. We used 10 % (*w*/*w*) silicalite solution in 5 mM phosphate buffer, pH 7.4. This suspension was ultrasonicated for at least 30 min, and then, 0.2 μl of suspension was deposited onto the active zone of each pair of electrodes; afterwards, they were heated at 120 °C for 15 min. Before the membrane deposition, the potentiometric transducers were cooled to room temperature and washed with working buffer from unbound silicalite particles. The surfaces of pH-FET with the silicalite particles and without them are presented in Fig. 1.

### Creatinine Deiminase Adsorption on Silicalite

To produce the enzyme membrane, we used 10 % CD solution in 20 mM phosphate buffer (pH 7.4) with 10 % glycerol, 4 % lactitol, and 0.4 % DEAE-Dextran. To prepare the reference membrane, the same components were used, but 10 % BSA solution was taken instead of the enzyme solution. A drop of the enzyme solution (0.16 μl) was deposited on the silicalite-modified working surfaces of pH-FET, a drop of BSA solution (0.16 μl), on other working surfaces of pH-FET (reference membrane).

After immobilization, the transducers were air-dried for about 30 min and then washed from unbound components in the working buffer for about 20 min.

### Creatinine Deiminase Immobilization in GA Vapor

Another procedure was CD immobilization in saturated GA vapor. In this case, to produce bioselective membranes, the solution of 10 % CD, 10 % BSA, 10 % glycerol, 4 % lactitol, and 0.4 % DEAE-dextran in 20 mM phosphate buffer, pH 7.4, was used. The solution for reference membrane was prepared in the same way, except that CD was replaced by BSA. After deposition of both solutions (0.1 μl each) onto the working surfaces of pH-FETs, the latter were placed in saturated GA vapor for 10–20 min and then air-dried for 15 min at room temperature. Next, the transducers were submerged into the working buffer for 20–30 min to wash off the unbound enzyme and GA excess.

### Measurement Procedure

Measurements were carried out in the 5 mM potassium-phosphate buffer solution (KH_2_PO_4_-NaOH), pH 7.4, with intensive stirring at room temperature. The biosensor and Ag/AgCl reference electrode were placed into an open measuring cell of volume 1.5 ml. The creatinine concentration in the working cell was obtained by an addition of aliquots of stock solution. The values of biosensor responses were calculated after reaching steady-state. After obtaining each response, the biosensor was washed from the products of reaction by changing working buffer (three times with 2 min intervals).

Non-specific changes in the output signal associated with fluctuations of temperature, medium pH, and applied voltage were compensated by using differential mode, i.e., measurement of the difference between the signals from two pH-FET electrodes (with enzyme and referent membranes), placed on the same transducer. All experiments were performed at least in three series.

## Results and Discussion

### Synthesis and Characterization of Silicalite

**Fig. 2:**
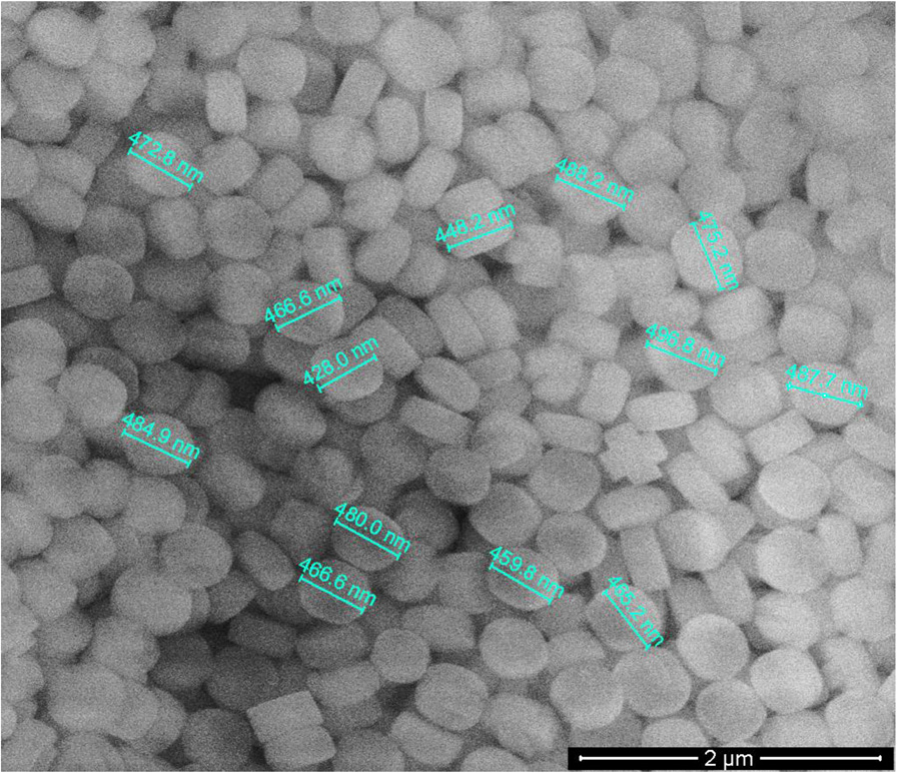
Scanning electron microscope image of silicalite

The first stage of our work was the silicalite particles synthesis and characterization. To synthesize silicalite, we used 1TPAOH:4TEOS:350H_2_O. Tetraethoxysilane (TEOS, 95 %) was used as a silica source and tetrapropylammonium hydroxide (TPAOH, 25 %)—as a template. By hydrolyzing TEOS with TPAOH solution, a clear homogeneous solution was obtained under stirring at room temperature for 6 h. Then, the resulting solution was kept in an oven for 18 h at 125 °C. The material, which did not react, was removed from the solution by centrifugation, washed with deionized water, and dried at 80 °C. The silicalite particles obtained were analyzed with a scanning electron microscope (Fig. 2).

**Fig. 3:**
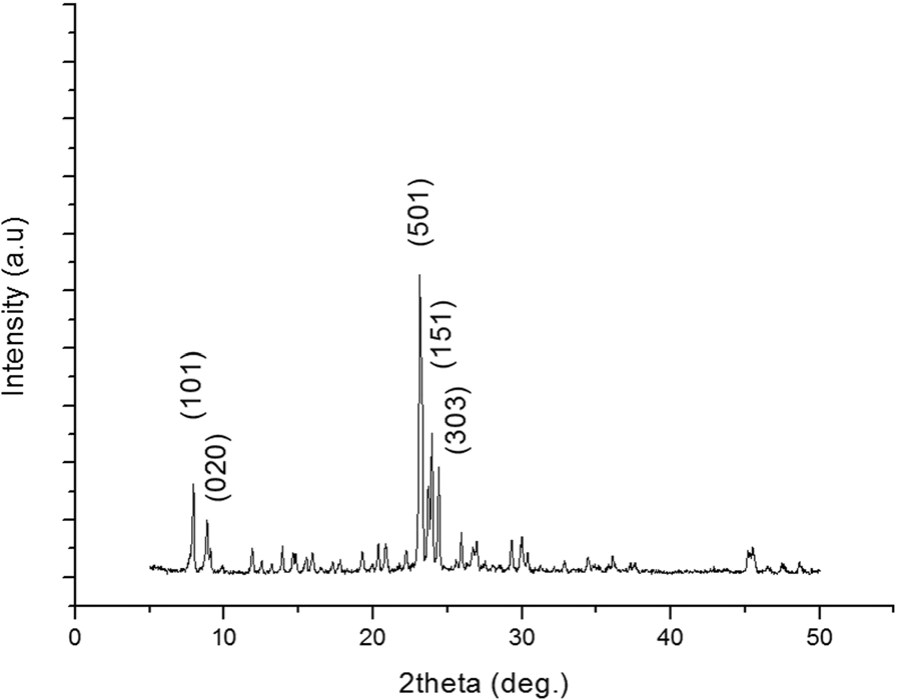
XRD spectrum of silicalite

Given the results obtained with an electron microscope (Fig. 2) were shown that the silicalite particles were approximately 470 nm in size, the data of X-ray diffraction (XRD) presented in Fig. 3 were contradictory with results of electron microscope. In Fig. 3, XRD peaks should be identified and attributed to a crystallographic structure. The synthesized silicalite has five distinct peaks as (101), (020), (501), (151), and (303) which were indicating the presence of silicalite structure [24].

But silica particles are polycrystalline, and their size are very different from that of isolated crystallites. Crystallite size can be calculated from full width half maximum, but at our size of silicalite as 470 nm, Scherrer’s equation underestimates the crystal size. As an example, Song et al. synthesized eight silicalite samples with different crystal sizes such as 20, 25, 39, 58, 74, 149, 300, and 1000 nm. They compared the particle sizes of the crystals using two different methods: from SEM images and from Scherrer’s equation [25]. They observed that the crystal sizes calculated from Scherrer’s equation were not consistent with the crystal sizes observed from SEM images, and the results were underestimating the crystal size especially for large crystals like 149, 300, and 1000 nm. For that reason, in our study, the size of silicalite was given from SEM images.

Additionally, for surface area of the samples, nitrogen adsorption–desorption isotherms were obtained. The surface area of the sample was 448 m^2^/g using multipoint BET method.

### Analytical Characteristics of CD-Based Biosensors

When elaborating biosensors for creatinine determination, the enzyme сreatinine deiminase was used. These biosensors function due to the enzymatic reaction:

Creatinine deiminase

Creatinine + H_2_O → *N*-methylhydantoine + NH_4_^+^

The pH changes induced due to creatinine hydrolysis are proportional to the substrate concentration in the tested solution and are registered by the pH-sensitive field-effect transistors with corresponding enzymatic membranes.

**Fig. 4:**
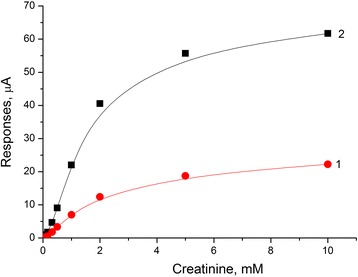
Calibration curves of creatinine biosensors: covalent cross-linking in GA vapor (*1*) and adsorption on silicalite (*2*). Measurements were carried out in 5 mM phosphate buffer solution, pH 7.4, at room temperature

Two types of biosensors were prepared using enzyme CD. The first was based on the enzyme adsorption on the transducer surface covered with a silicalite layer, whereas the second—on the traditional method of enzyme immobilization in glutaraldehyde vapor. The study was focused on comparison of the basic analytical characteristics of biosensors, in which different methods of immobilization were utilized. The calibration curves of creatinine determination in the working buffer were plotted for both types of biosensors (Fig. 4).

It was shown that application of adsorption on silicalite allowed an increase in the sensitivity to creatinine by two to three times, a three- to fourfold decrease in times of response and recovery, and a twofold lower minimum limit of determination (Table 1), as compared with a covalent cross-linking in GA vapor. Here, the linear dynamic range remained the same for both options of immobilization and ranged from 0 to 2 mM.

**Table 1:** Characteristics of creatinine biosensor based on different types of enzyme immobilization

Key parameters	Covalent CD cross-linking in GA vapor	CD adsorption on silicalite
Minimum limit of determination, μM	10	5
Response time, s	240	90
Recovery time, s	300	80
Sensitivity, μА/mМ	6.68	18.06

Therefore, the method of enzyme immobilization by adsorption on silicalite-modified electrodes was chosen as the most appropriate for the biosensor creation; for this reason, the further study was carried out using only the membrane based on CD adsorption on silicalite.

### Biosensor Stability Under Continuous Operation and Storage

**Fig. 5:**
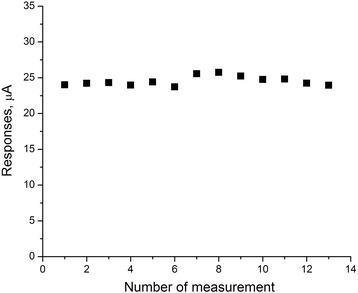
Signal reproducibility of Sil/СD-based biosensor. Measurements were carried out in 5 mM phosphate buffer solution, pH 7.4, at room temperature. Creatinine concentration was 1 mM

As the signal reproducibility and storage stability are the most important working characteristics of biosensors, it was necessary to check these options for the biosensor based on a new method of immobilization and CD adsorption on silicalite. To determine signal reproducibility, the biosensor responses to 1 mM creatinine were measured over one working day. During intervals between measurements, the biosensors were kept in continuously stirred buffer solution at room temperature. As seen from Fig. 5, the responses of Sil/CD biosensor were highly reproducible. The relative standard deviation (RSD) for creatinine determination was 2.6 %.

To investigate the storage stability, a special buffer with the addition of stabilizers and preservatives (10 mM phosphate buffer, pH 7.4 + 1 mM EDTA + 1 mM dithiothreitol + 0.1 % NaN_3_) was used.

The biosensors were stored in this buffer at 4 °C. On the first day after adsorption of the enzyme and reference membranes on the transducer surface covered with a layer of silicalite, the biosensor responses to 1 mM creatinine were measured in 5 mM phosphate buffer, pH 7.4. The responses of biosensors with initial activity were taken as 100 %.

**Fig. 6:**
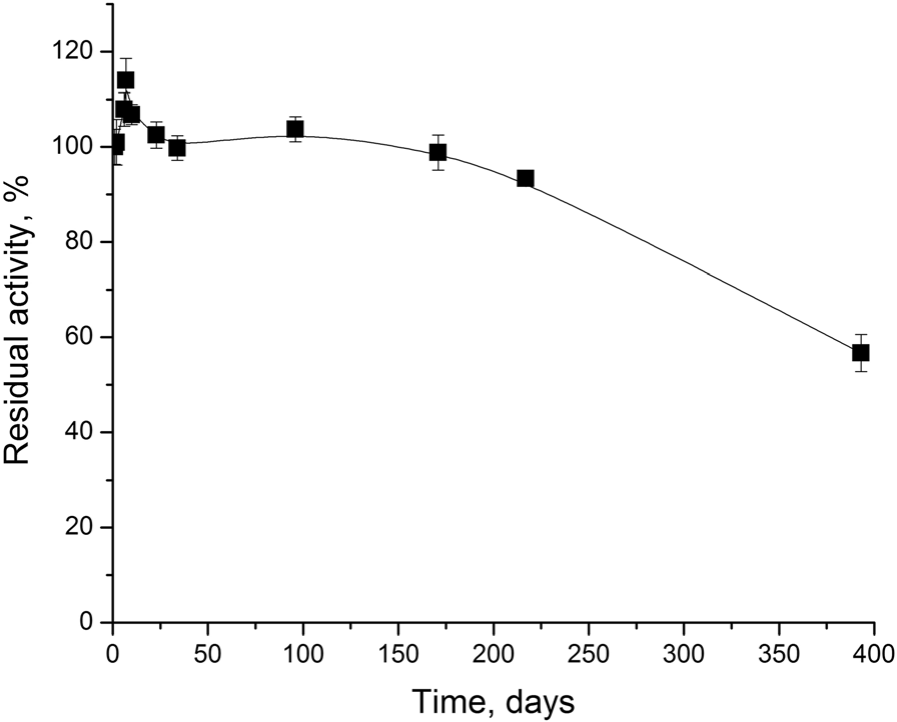
Storage stability of the Sil/CD-based biosensor. Measurements were carried out in 5 mM phosphate buffer solution, pH 7.4, at room temperature. Creatinine concentration was 1 mM

Subsequent measurements were carried out after certain time intervals. It was shown that after more than 1-year long storage, the activity of CD adsorbed on silicalite decreased by only 43.3 % (Fig. 6). This is very high characteristic, considering that CD is known as an unstable enzyme. It can be an effect of silicalite as a carrier at the immobilization, due to zeolites high tolerance to microorganisms.

### Optimization of Biosensor for Blood Serum Analysis

The next task was to test the developed biosensor in real biological samples, namely, blood serum of patients with renal failure, where the creatinine concentration is about 1000 μM. The composition of blood serum is complex; therefore, the analysis of its samples is quite complicated. Blood serum contains several buffer systems, amino acids, electrolytes, carbohydrates, enzymes, lipoproteins, proteins, etc. Especially, high are the concentrations of sodium chloride (137–144 mM) and protein (7.5 % on average).

**Fig. 7:**
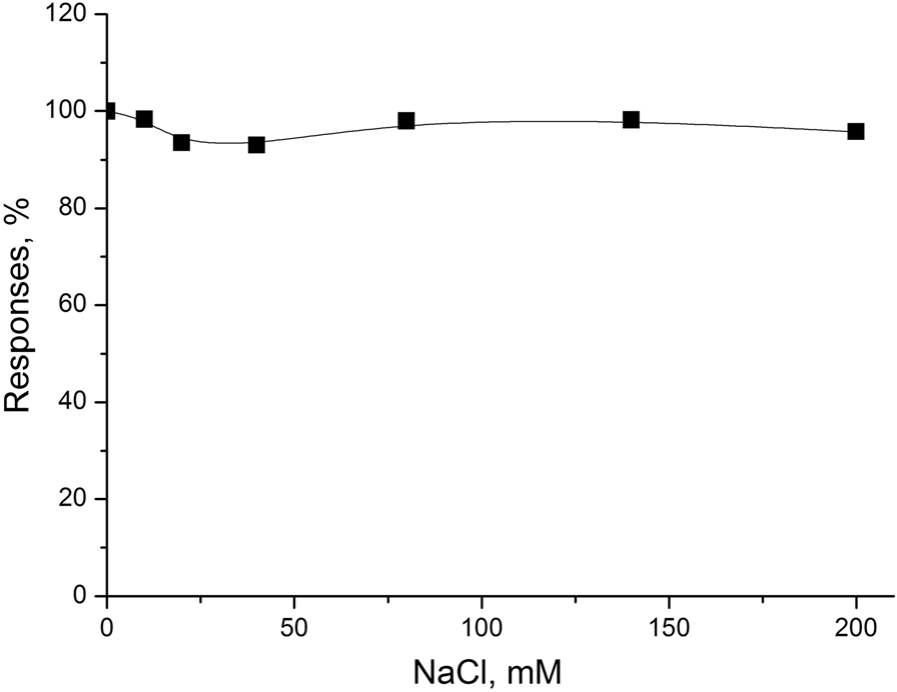
Dependence of response of Sil/CD-based biosensor on NaCl concentration. Measurements were carried out in 5 mM phosphate buffer solution, pH 7.4, at room temperature. Concentration of creatinine was 1 mM

To this end, the biosensor responses to 1 mM creatinine were measured at the sodium chloride concentrations ranged from 0 to 200 mM. It was shown that the value of responses distinguished by 2 to 7 %. Since the 1:20 blood serum dilution and differential mode of measurements were planned to be used, an influence of NaCl could be neglected (Fig. 7).

**Fig. 8:**
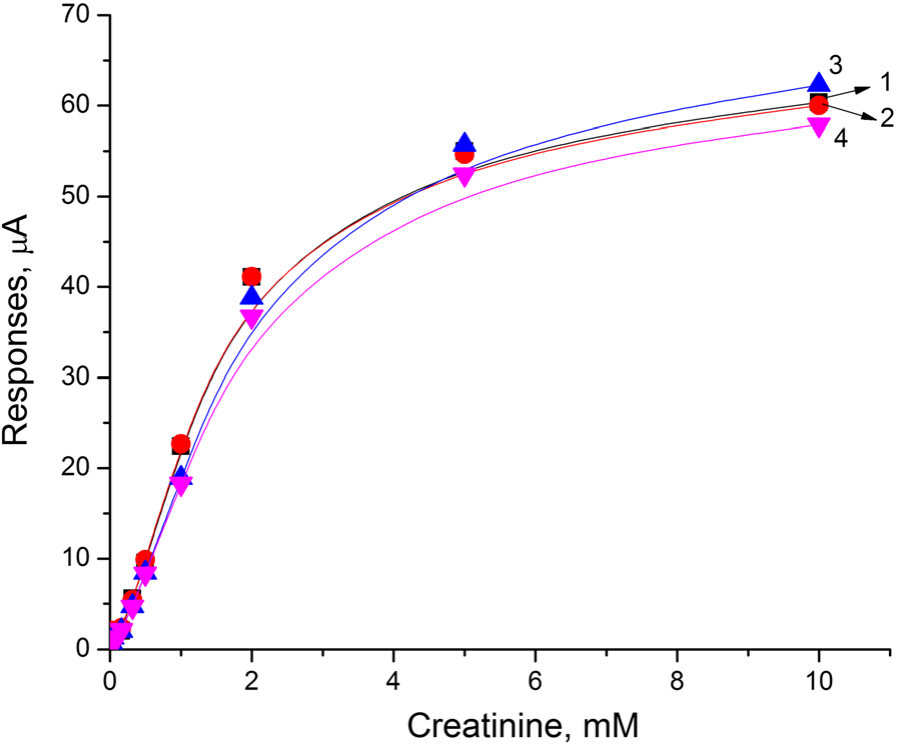
Calibration curves for creatinine detection in presence of protein at various concentrations: 0 % (*1*), 0,1 % (*2*), 0,5 % (*3*), and 1 % (*4*). Measurements were carried out in 5 mM phosphate buffer solution, pH 7.4, at room temperature

Considering possible variants of blood serum dilution from 1:10 tо 1:20, BSA was also added to the working cell in concentrations up to 1 %; no signal to BSA was observed, and no significant influence of BSA on calibration curves of the biosensor was found (Fig. 8).

Thus, the presence of pre-diluted macromolecular BSA fraction in the buffer does not lead to a significant non-specific response and faintly affects its value.

The developed Sil/CD-based biosensor has excellent analytical characteristics, high storage stability, and selectivity towards interferences. Therefore, in the future, it can be successfully used in the analysis of blood serum of patients with kidney disease.

## Conclusions

A possibility of efficient use of creatinine deiminase adsorption on the surface of silicalite for the creation of novel biosensor was investigated. The procedure of enzyme immobilization is simple, rapid, and non-toxic. The conditions of creatinine deiminase adsorption on silicalite were optimized. The working parameters of the created Sil/CD-based biosensor were investigated. The developed method of enzyme adsorption on silicalite was compared with the common immobilization procedure in glutaraldehyde vapor. It was shown that the creatinine-sensitive biosensor, created by covalent cross-linking in GA vapor, was threefold less sensitive to creatinine and had three- to fourfold longer response time and twice the minimum limit of determination. The creatinine detection range of the developed biosensor was determined to be 0.005–2 mM. The created biosensor with adsorbed creatinine deiminase was characterized by good reproducibility (RSD = 2.6 %) and storage stability (about 13 months). It was found that sodium chloride at concentrations 0–200 mM and BSA at concentrations 0.1–1 % had no significant impact on the biosensor responses. This fact is crucial for the further application of the biosensor for creatinine analysis in blood serum. The method of enzyme adsorption on silicalite has such advantages as quickness, simplicity, the absence of toxic chemical reagents, and good reproducibility. The developed biosensor based on this method can be used for monitoring creatinine in clinical practice.
